# Combination of IFNα and poly-I:C reprograms bladder cancer microenvironment for enhanced CTL attraction

**DOI:** 10.1186/s40425-015-0050-8

**Published:** 2015-03-24

**Authors:** Ravikumar Muthuswamy, Liwen Wang, Jamie Pitteroff, Jeffrey R Gingrich, Pawel Kalinski

**Affiliations:** Departments of Sugery, University of Pittsburgh, Pittsburgh, PA 15213 USA; Department of Urology, University of Pittsburgh, Pittsburgh, PA 15213 USA; Department of Infectious Diseases and Microbiology, University of Pittsburgh, Pittsburgh, PA 15213 USA; Department of Immunology, University of Pittsburgh, Pittsburgh, PA 15213 USA; University of Pittsburgh Cancer Institute, Pittsburgh, PA 15213 USA; Department of Surgery, University of Pittsburgh, Hillman Cancer Center, UPCI Research Pavilion, Room 1.46b, 5117 Center Avenue, Pittsburgh, PA 15213 USA

**Keywords:** Tumor microenvironment, Immunomodulation, Chemokines, BCG, TLR3, Poly-I :C, IFNα, Bladder cancer, Effector T cells, Regulatory T cells

## Abstract

**Background:**

BCG is a prototypal cancer immunotherapeutic factor currently approved of bladder cancer. In attempt to further enhance the effectiveness of immunotherapy of bladder cancer and, potentially, other malignancies, we evaluated the impact of BCG on local production of chemokines attracting the desirable effector CD8^+^ T cells (CTLs) and undesirable myeloid-derived suppressor cell (MDSCs) and regulatory T(reg) cells, and the ability of bladder cancer tissues to attract CTLs.

**Methods:**

Freshly resected bladder cancer tissues were either analyzed immediately or cultured *ex vivo* in the absence or presence of the tested factors. The expression of chemokine genes, secretion of chemokines and their local sources in freshly harvested and *ex vivo*-treated tumor explants were analyzed by quantitative PCR (Taqman), ELISAs and immunofluorescence/confocal microscopy. Migration of CTLs was evaluated *ex vivo*, using 24-transwell plates. Spearman correlation was used for correlative analysis, while paired Students *T* test or Wilcoxon was used for statistical analysis of the data.

**Results:**

Bladder cancer tissues spontaneously expressed high levels of the granulocyte/MDSC-attractant CXCL8 and T_reg_-attractant CCL22, but only marginal levels of the CTL-attracting chemokines: CCL5, CXCL9 and CXCL10. Baseline CXCL10 showed strong correlation with local expression of CTL markers. Unexpectedly, BCG selectively induced only the undesirable chemokines, CCL22 and CXCL8, but had only marginal impact on CXCL10 production. In sharp contrast, the combination of IFNα and a TLR3 ligand, poly-I:C (but not the combinations of BCG with IFNα or BCG with poly-I:C), induced high levels of intra-tumoral production of CXCL10 and promoted CTL attraction. The combination of BCG with IFNα + poly-I:C regimen did not show additional advantage.

**Conclusions:**

The current data indicate that suboptimal ability of BCG to reprogram cancer-associated chemokine environment may be a factor limiting its therapeutic activity. Our observations that the combination of BCG with (or replacement by) IFNα and poly-I:C allows to reprogram bladder cancer tissues for enhanced CTL entry may provide for new methods of improving the effectiveness of immunotherapy of bladder cancer, helping to extend BCG applications to its more advanced forms, and, potentially, other diseases.

**Electronic supplementary material:**

The online version of this article (doi:10.1186/s40425-015-0050-8) contains supplementary material, which is available to authorized users.

## Background

Bladder cancer, originating from the transitional cells of the bladder urothelium, accounts for an estimated 72,570 new cases and 15,210 deaths in the US. Though bladder cancer is highly treatable if found early, it becomes increasingly difficult to treat at later stages. Intravesicular BCG administration has been the standard therapy for treatment of bladder cancer, but its effectiveness is limited only to superficial bladder cancers [1-4].

Tumor infiltration with effector CD8^+^ T cells (CTL) has been associated with good prognosis in various cancers [5-18], therefore immunotherapies able of enhancing intra-tumoral CTL levels may be also effective for invasive bladder cancers. Previous studies by us [19] and others [10,20-25] have shown that intra-tumoral expression of chemokines regulate local levels of CTL infiltration, suggesting that T cell-targeting immunotherapies can benefit from modulating tumor-associated chemokine microenvironments in order to enhance local CTL infiltration.

Since myeloid-derived suppressor cell (MDSCs) and regulatory T_(reg)_ cells are both known to protect tumors from CTL-mediated elimination and to promote tumor growth, ideal immunotherapies should be able to selectively enhance tumor production of CTL-attracting chemokines, without enhancing local levels of CXCL8, CXCL12 and CCL22, the chemokines mediating local attraction of MDSC and T_reg_ to tumors [26-30]. Prompted by the above considerations, we evaluated the ability of BCG and alternative adjuvants to reprogram local chemokine milieu in bladder cancer to enhance the overall magnitude of local production of CTL-attracting chemokines in relation to MDSC/T_reg_-attractants. Unexpectedly, we observed that BCG, used alone, not only failed to enhance local expression of CTL-attracting chemokines, CCL5 and CXCL10, but selectively enhanced MDSC- and T_reg_-attracting chemokines, CCL22 and CXCL8. These undesirable side-effects could be reversed by the combination of IFNα and poly-I:C (TLR3 ligand), raising the possibility of enhancing the effectiveness of the BCG-based and other forms of immunotherapy of bladder cancer and potentially other malignancies.

## Results

### Bladder cancer tissues spontaneously produce MDSC- and T_reg_-attracting CXCL8 and CCL22, but not effector T cell-attracting chemokines

**Figure 1:**
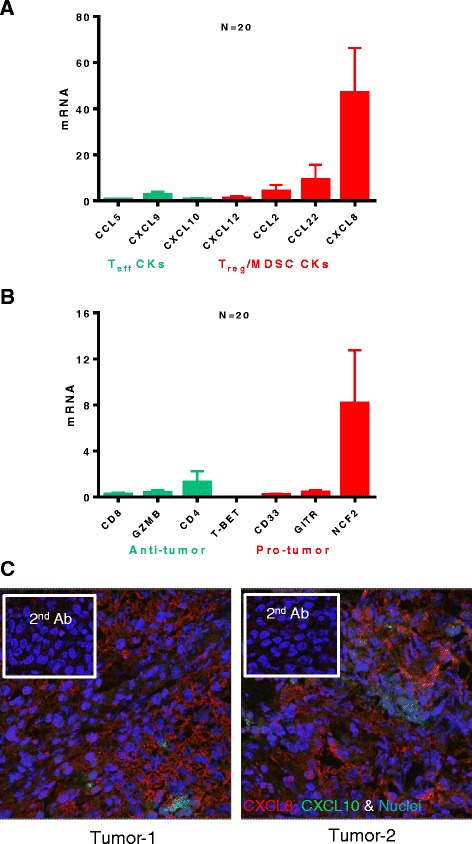
**Bladder cancer tissues spontaneously express high levels of MDSC/T**_**reg**_ **- attracting chemokines CCL22 and CXCL8, but only marginal levels of CTL- attractants, CCL5, CXCL9 and CXCL10. A**. Spontaneous chemokine mRNA expression by bladder tumors (N = 20 different patients) by real-time PCR analysis (Taqman). **B**. Spontaneous mRNA expression analysis for markers of immune filtrate in bladder tumors (N = 20 different patients) by real-time PCR analysis (Taqman). The levels of chemokine and immune filtrate markers were normalized to HPRT1 mRNA (housekeeping gene). **C**. Confocal analysis for CXCL8 (Red), CXCL10 (Green) protein expression in 2 representative bladder tumors. Nuclei were stained with Sytox orange (Blue) and specificity controls (secondary antibody only) are shown in insets.

In order to evaluate the spontaneous chemokine expression and determine the baseline chemokine production pattern in bladder tumors, we isolated RNA from resected tumors of bladder cancer patients (N = 20) and performed real-time PCR (Taqman) analysis for the chemokines previously implicated in CTL or MDSC/T_reg_ attraction to tumor lesions [26-30]. We observed that bladder tumors uniformly expressed only low levels of CTL attracting chemokines: CCL5, CXCL9, and CXCL10 (respective ligands for CTL-expressed CCR5 and CXCR3 [19]). In a striking contrast to the above CTL-attractants, the chemokines implicated in attracting MDSCs and T_regs_, CCL2 (MCP-1), CCL22 (MDC) and CXCL8 (IL-8) were highly expressed chemokines (Figure 1A), with CXCL8 uniformly expressed at the highest levels. The prevalence of high CCL22 and CXCL8 in bladder tumors at baseline is consistent with previous studies [31-33] which showed the abundance of these undesirable chemokines, particularly in patients with poor prognosis. High intra-tumoral expression of CXCL8 was confirmed by confocal analysis of tumor samples (Figure 1C). Taqman analysis for different markers of immune infiltrate (Figure 1B), using primers for CD4, CD8, GZMB and T-bet (effector cell markers), CD33 (myeloid marker), GITR (Treg marker) and NCF2 (myeloid or neutrophil marker) revealed that NCF2 expression was dominant, corresponding to the high IL-8 levels (see below).

### Intra-tumoral expression of CCL5, CXCL9, and CXCL10 shows a strong correlation with CTL markers CD8 and Granzyme B

**Figure 2:**
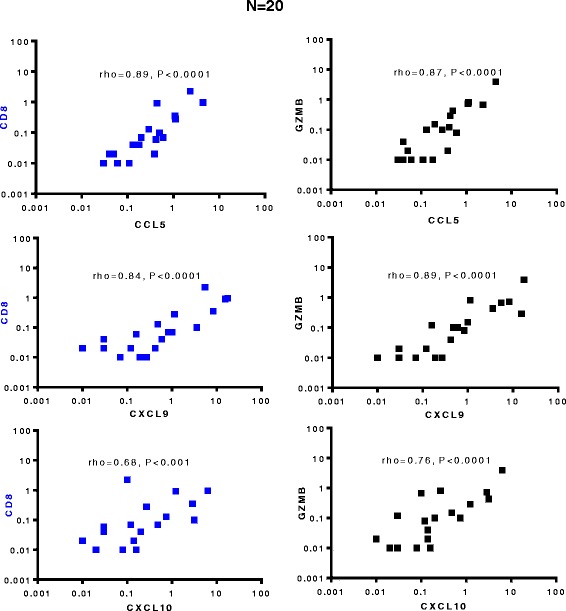
**CCR5- and CXCR3-binding chemokines (CCL5, CXCL9 and CXCL10) show high correlation with CTL markers in bladder cancer tissues.** Spearman (Rho) analysis of the correlations between the spontaneous expression (mRNA) of CTL markers (CD8 and Granzyme B) and CTL attracting chemokines (CCL5, CXCL9 and CXCL10). Values on the scale are log10 transformations of relative mRNA levels for each of the markers were evaluated using real time PCR (Taqman).

In order to test if the spontaneous expressed nominal CTL-attracting chemokines indeed predict local CTL infiltration in bladder cancer tissues, we attempted to correlate intra-tumoral mRNA expression of CCR5 ligands and CXCR3 ligand (CCL5, CXCL9 and CXCL10) with Intra-tumoral mRNA expression of CTL markers. As shown in Figure 2, Spearman correlative analysis of the chemokines and CTL markers revealed strong correlation between CD8 and Granzyme B with CCL5, CXCL9 and CXCL10. As expected, neither of these markers was correlated with the local expression of a T_reg_-attractant, CCL22 (data not shown). The levels of IL-8 expression were strongly correlated with NCF2 expression (neutrophil marker; Additional file 1: Figure S1).

### BCG treatment of bladder tumors up regulates CXCL8, CCL22 expression, but not CXCL10

**Figure 3:**
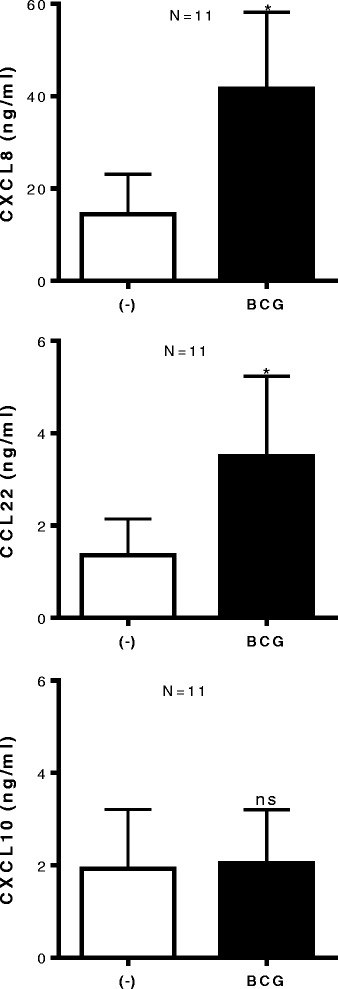
**BCG exposure of bladder tissues does not induce CXCL10, but further enhances tumor production of MDSC/T**_**reg**_ **- attracting CCL22 and CXCL8.** Bladder tumor biopsies were cultured in the absence or presence of BCG (2 × 10^6^ CFU) for 24 hours. CXCL10, CCL22 and CXCL8 proteins in tumor supernatants were measured by ELISA and expressed as ng/ml. The results were evaluated using two- tailed, paired Student’s *t* test. Statistically significant differences between groups are highlighted by * (P < 0.05). NS-Not significant.

Using CXCL10 as a representative of CTL-attracting chemokines, involved in attraction of CXCR3^+^ CTL implicated with good prognosis for cancer patients [7,11,12,15], we tested whether BCG treatment can enhance intra-tumoral production CXCL10. Unexpectedly, we observed that, while BCG strongly up regulated the secretion of CXCL8 (P < 0.05) and CCL22 (P < 0.05) by *ex vivo*-treated bladder cancer explants (n = 11 patients), it did not enhance CXCL10 secretion (Figure 3).

### Combination of IFNα + poly-I:C reverses the BCG-driven enhancement of undesirable chemokines

Since we have previously demonstrated that the combination of IFNα with poly-I:C effectively up regulated CTL chemokines in colorectal cancer tissues [19], we tested whether their addition to BCG can enhance its effectiveness. In a preliminary set of experiments we used an *in vitro* model system involving TS4 bladder cancer cells, blood isolated monocytes and fibroblasts (Additional file 1: Figure S3) to directly compare multiple combinatorial adjuvants in a single experiment, without the limitation imposed by the amount of bladder cancer tissues available from resections and their variability between different patients. In accordance with the data from our bladder cancer explant cultures, BCG alone was completely ineffective in promoting CXCL10 secretion in such cell cultures. In contrast, we observed strong synergy between IFNα and poly-I:C in promoting CXCL10 secretion, both in the absence and in the presence of BCG (Additional file 1: Figure S3). Importantly, neither the combination of BCG with poly-I:C nor the combination of BCG with IFNα was effective, which may explain the limited effectiveness of that later combination in the recently-completed clinical trial [4,34,35].

**Figure 4:**
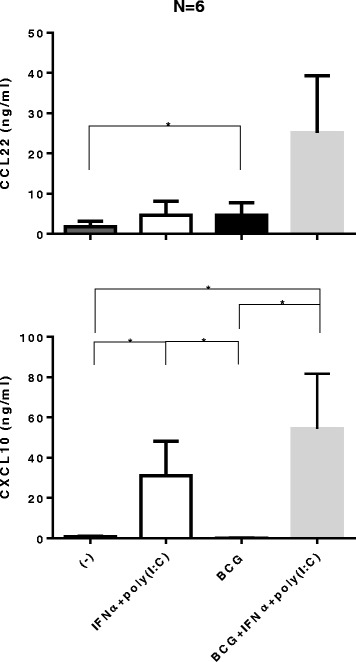
**Combination of IFNα with poly-I:C is a powerful inducer of CXCL10 in bladder cancer lesions in the absence or presence of BCG.** Bladder tumors biopsies were cultured for 24 hours in the absence or presence of 10,000 units IFNα + 20 μg/ml specific poly-I:C, with or without BCG (2 × 10^6^ CFU). The levels of CXCL10 and CCL22 in tumor supernatants were measured by specific ELISAs. The results were evaluated using two- tailed, paired Wilcoxon Test. Statistically significant differences between groups are highlighted by * (P < 0.05).

In accordance with these observations, our experiments performed in the tumor tissue explant model (n = 6 patients), demonstrated that in contrast to BCG, the combination of IFNα and poly-I:C strongly elevated tumor secretion of CXCL10. The combination of BCG with IFNα + poly-I:C resulted in only marginal or no further enhancement of CXCL10 secretion, but was associated with the undesirable elevation of CCL22 (Figure 4).

### IFNα + poly-I:C-treated tumors show enhanced attraction of effector CD8^+^ cells

**Figure 5:**
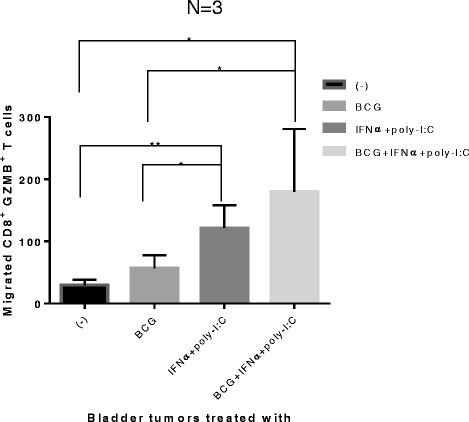
**Bladder cancer lesions exposed to IFNα + poly-I:C or BCG + IFNα + poly-I:C show strongly-enhanced CTL-attracting activity.** Day 6 effector Granzyme B^+^/CD8^+^ T cells (induced by SEB-loaded LPS + IFNγ-matured DC [36,37]) were harvested and allowed to migrate for 2 hours towards the supernatants from the differentially-treated bladder cancer tissues in 24 trans-well system. Migrated cells in the bottom chamber were harvested and stained for CD8, Granzyme B. The counts of Granzyme B^+^ CD8^+^ T cells were analyzed by FACS. The results were evaluated using two- tailed, paired Student’s *t* test. Statistically significant differences between groups are highlighted by * (P < 0.05) or ** (P < 0.01).

To test whether the modified chemokine production patterns in BCG- and IFNα + poly-I:C-treated bladder cancer tissues result in their differential ability to attract CTLs, supernatants of the differentially-treated bladder cancer tissues were tested for their ability to attract *ex vivo*-induced effector CD8^+^ T cells (pre-activated by SEB-loaded LPS + IFNγ matured DCs, capable of high IL-12 production [36,37]). As expected, bladder cancer tissues (n = 3 patients) treated with IFNα + poly-I:C significantly attracted more of the effector CD8^+^ T cells than untreated or BCG alone treated tumors (Figure 5). The combination of BCG with IFNα + poly-I:C didn’t further increase the CTL attraction. These data indicate that BCG by itself is insufficient to reprogram the bladder cancer-associated chemokine environment for enhanced CTL attraction, but that such goal can be achieved by the combination of BCG with (or its replacement by) IFNα plus poly-I:C.

## Discussion

BCG is a prototypal cancer immunotherapeutic factor which has been widely demonstrated to be effective in the treatment of superficial bladder cancer [1-4]. In attempt to further enhance the effectiveness of the immunotherapy of bladder cancer, including its more advanced stages, and potentially other malignancies, we evaluated the impact of BCG on local production of the chemokines attracting the desirable effector CD8^+^ T cells and undesirable MDSCs and T_reg_ cells.

We observed that bladder cancer tissues spontaneously expressed high levels of T_reg_- and MDSC- recruiting chemokines (CCL2, CCL22 and CXCL8, respective ligands for CCR2, CCR4 and CXCR1/2), but only low levels of CTL- attracting chemokines (CCL5, CXCL9-10; respective ligands for CCR5 and CXCR3), suggesting that the chemokine imbalance can contribute to the pathogenesis of bladder cancer and may limit the effectiveness of its immunotherapies. Unexpectedly, despite the documented beneficial role of BCG in bladder cancer, our data indicate that its ability to modify the microenvironment of bladder cancer *ex vivo* is largely limited to the enhancement of local production of T_reg_- and MDSC-attracting chemokines, CCL22 and CXCL8, without inducing CXCL10 or facilitating CTL attraction. Our current data is consistent with the previous observations that bladder cancer tissues do not produce the desirable chemokine CXCL10 within the first week of BCG treatment, although can produce this factor after 3 weekly doses of BCG [38]. They are also consistent with the previous observations that bladder cancer lesions typically produce high levels of CCL22 and CXCL8 at baseline [31-33,39,40], which may be further amplified by treatment of patients with BCG and particularly by treatment with by BCG combined with chemotherapy [32], constituting the undesirable side-effect of treatment [33]. Since intratumoral expression of CXCL8 has been shown to be a negative prognostic marker [31], these observations highlight a potential for targeting the bladder cancer-associated chemokine microenvironments to improve the outcomes of BCG-treatment and chemotherapy of this cancer type.

This selective activity of BCG may result from its dominant pattern of TLR2-mediated signaling [41], which has been shown to induce significantly less type-1 interferons compared to other TLR ligands [42,43]. In accordance with this possibility, our observations suggests that the combination of IFNα with poly-I:C (a TLR3 ligand) or BCG + IFNα + poly-I:C (although neither BCG + IFNα nor BCG + poly-I:C) are highly effective in enhancing intra-tumoral production of CXCL10 in bladder cancer tissues and promoting CTL infiltration. Interestingly, both these effective combinations showed at least partial selectivity in inducing CXCL10 (rather than CCL22) in cancer tissues. Our current experiments aim to determine if the additional inclusion of COX inhibitors (which proved to be effective in suppressing local production of CCL22 in metastatic colon cancer lesions [19]) with the currently-evaluated BCG + IFNα + poly-I:C combination will further potentiate its effectiveness by suppressing COX-2 dependent production of CCL22, CXCL8 and boosting induction of CXCL10 and other CTL-attracting factors.

## Conclusion

Our current data suggest that the suboptimal ability of BCG to reprogram cancer-associated chemokine environments may represent a factor limiting its clinical activity, especially in more advanced stages of bladder cancer, and may represent an area of additional therapeutic intervention. Our identification of the combination of IFNα and poly-I:C, as factors which may supplement (or replace) BCG in effective reprogramming of cancer tissues for enhanced CTL entry, may help to improve the effectiveness of immunotherapy of bladder cancer and enhance the applications of BCG to its more advanced forms, as well as other malignancies or other disease states (such a mycobacterial infections), where the current BCG-based therapeutic regimens are not effective.

## Methods

### *Ex vivo* culture of bladder tumor explant tissues

Tumor from patients was obtained by informed consent under the IRB approved protocol UPCI 86–022. Patient clinical characteristics and sets of patients used for specific types of experiments are given in Table 1 below.

**Table 1:** **Bladder cancer patients**

	**Patient number N (%)**			
	**Total**	**qPCR analysis**	**ELISA analysis**	**Chemotaxis**
**Patients**	**23(100)**	**20(100)**	**11(100)**	**3(100)**
Male	15(65)	14(70)	6(55)	1(33)
Female	8(35)	6(30)	5(45)	2(67)
**Tumor stage:**				
Stage 0	4(17)	4(20)	2(18)	
Stage 0is	1(4)	1(5)		
Stage 1	6(26)	5(25)	3(27)	1(33)
Stage 2	2(9)	2(10)	1(9)	
Stage 3	8(35)	6(30)	4(36)	2(67)
Stage 4	2(9)	2(10)	1(9)	
**Prior treatment:**				
None	15(65)	13(65)	7(64)	2(67)
BCG	5(22)	5(25)	2(18)	
Other treatment	3(13)	2(10)	2(18)	1(33)

Using a 2 mm biopsy punch knife, uniform 2 mm cubes of resected tumor tissue were made. Tumor explants were assorted as 3 × two mm cubes/wells in 48 well plate respectively and cultured in IMDM plus 10% FBS, either untreated or treated with 2 × 10^6^ CFU of BCG (TICE® BCG, Schering Plough, NJ) and/or 10,000 units of IFNα (Merck, NJ), 20 μg/ml of poly-I:C (Sigma Aldrich, St. Louis, MO). Tumor tissue biopsies were harvested at 0 and 24 hours for mRNA and confocal microscopy analysis and culture supernatants were harvested at 24 hours for ELISA.

### Bladder cancer cell line-macrophage-fibroblast co-culture system

In order to compare multiple combinatorial adjuvants in a single experiment, we established a model system involving 1 × 10^5^ each of TS4 (bladder cell line; ATCC, Manassas, VA), blood isolated monocytes (isolated by CD14 microbeads; Miltenyi Biotech.) and fibroblasts (Life technologies, Grand Island, NY) in 48 well plates. The cells were treated with the indicated permutation combinations of celecoxib (10 μM, Biovision, Milpitas, CA), IFNα (10000 units/ml), poly-I:C (20 μg/ml) and BCG (2 × 10^6^ CFU) for 24 hours and supernatants were harvested for ELISA analysis of various chemokines. In preliminary experiments, we used macrophages generated from healthy donors’ monocytes in 6 day-long GM-CSF-supplemented cultures, to directly compare the chemokine-modulating impact of increasing doses of BCG in the same experiment. As shown in Additional file 1: Figure S2, different doses of BCG (0.4, 2 and 10 × 10^6^ CFU), all induced high levels of CXCL8, but not CXCL10.

### Taqman analysis of mRNA expression in tumor

Tumor biopsies were placed into lysing Matrix E tubes (MP Biologicals, Solon, OH), containing RLT buffer (RNAeasy kit, Qiagen, Valencia, CA), agitated in FP120 homogenizer (MP Biologicals). Debris-free supernatants from the lysis matrix tubes were transferred into new tubes and total RNA was extracted using the RNAeasy kit (Qiagen,MA). 1 μg of RNA extracted by above method was used for cDNA synthesis using Quanta biosciences synthesis kit and 25–50 ng of the resulting cDNA was used to do mRNA expression analysis by Taqman, using primers and equipment (Step One Plus system) from (Life Technologies, Long Island, NY).

### Confocal microscopy analysis of tumor sections

Tumor biopsies were embedded in OCT medium containing cryomolds and immediately frozen in 2-methyl-butane. 5 μm frozen sections of the tissues were made using the cryostat and layered on superfrost® plus slides (Thermo Scientific, Rockford, IL). The slides were fixed in 4% para-formaldehyde for 15 minutes, washed and blocked for 60 minutes at room temperature. The slides were then stained for 3 hours at room temperature (RT) with antibodies for CXCL10 (ab9807) and CXCL8 (ab18672), both from Abcam, Cambridge, MA. The slides were washed 5 times with 1 × PBS and incubated with secondary antibodies anti-rabbit-Alexa 488, anti-mouse-Alexa 647 (both from Cell Signal, Danvers, MA) and with nuclear dye Sytox orange (Invitrogen, Carlsbad, CA) for 30 minutes at RT. The slides were washed 5 times with 1 × PBS. Cover slips were mounted on the sections using prolong gold anti-fade solution (Life Technologies). Confocal analyses of stained slides were performed using a LEICA TCS SL DMRE Microsystems.

### ELISA analysis of chemokines in tumor *ex vivo* culture or in cell co-cultures

24 hour culture supernatants from tumor *ex vivo* culture were analyzed by ELISA for chemokine proteins expressed. ELISA primary and secondary antibody pairs for the analysis of CCL5, CXCL8 and CXCL10, protein secretion were purchased from Peprotech, Rocky Hill, NJ. ELISA plates (Corning Inc, Corning, NY) were coated overnight at RT with 100 μL of primary antibody at 1 μg/mL, followed by washing and blocking with PBS + 4% BSA for 1 hour. 50 μL of samples were added to the wells and incubated for 1 hr and subsequently washed and incubated with 50 μL of biotinylated secondary antibodies at 0.5 μg/mL for 1 hour. The plates were washed and incubated for 30 minutes with streptavidin-HRP conjugate (Pierce Biotechnology Inc, Rockford, IL), diluted 1:10000 in wash buffer (50 mM Tris, 0.2% Tween). The plates were washed and detected with 50 μL of TMB substrate (Pierce). Reactions were stopped with 2% H_2_SO_4_ and 450 nm absorbance was measured, using Wallac 1420 Victor 2 microplate Reader (Perkin Elmer, Waltham, MA).

### Generation of effector T cells

Naïve CD8^+^ T cells were purified from peripheral blood of normal donor using EasySep naïve CD8 enrichment kit (#19158, Stemcell Tech, Vancouver, Canada). Isolated naïve CD8^+^ T cells were stimulated with SEB (1 ng/ml) pulsed, LPS + IFNγ-matured DC for 6 days [36]. On 6th day, effector CD8^+^ T cells were harvested, cells counted, density-adjusted to 10^6^ cells per ml and used in chemotaxis assays.

### Chemotaxis

In a 24 trans-well plates with membrane with 5 μM pore size (#3421, Corning, CA), 2 × 10^5^ (200ul) effector CD8^+^ T cells were loaded onto top chambers and allowed to migrate for 2 hours [19,36,44] towards 400 μl of supernatants generated from 24 hour bladder cancer explant cultures. Migrated cells were harvested from the bottom chambers and stained for CD8 and Granzyme B (GZMB). Number of migrated GZMB positive effector CD8^+^ T cells were quantified by fixed 100 μl run on Accuri C6 (BD Biosciences, San Jose, CA).

### Statistical analysis

Spearman rank correlations (rho) between the mRNA of chemokine genes and cytotoxic T cell markers were calculated. Comparisons of continuous variables between groups were performed by 2 tailed, paired *t* test or paired Wilcoxon test using graphPad Prism 6 software. The values of P <0.05 were considered as significant.

## Additional file

Additional file 1: Figure S1. Intratumoral CXCL8 expression shows strong correlation with the neutrophil marker, NCF2. Spearman (rho) analysis of the correlation between intra-tumoral mRNA expression of CXCL8 (IL-8) with Intra-tumoral expression of NCF2 (Neutrophil marker). **Figure S2.** BCG dose titration analysis and its impact on chemokine secretion by macrophages. Macrophages, generated by culturing monocytes in presence of GMCSF for 6 days, were exposed to increasing doses of BCG (0.4, 2, 10 × 106 CFU) for 24 hrs. Secretion of CXCL8 and CXCL10 was measured by specific ELISAs. **Figure S3.** BCG combination with IFNα + poly-I:C reduces CXCL10 levels in co-cultures of bladder cancer cells, macrophages and fibroblasts. TS4(Bladder cell line) was co cultured with macrophages and Fibroblasts in ratio 1:1:1 and treated with different permutations of celecoxib, IFNα, poly-I:C, and BCG. CXCL10 secretion was measured in 24 hour supernatants by ELISA. CCL22 and CXCL8 were below detection levels.
