# Supplementary material for: Combination of IFNα and poly-I:C reprograms bladder cancer microenvironment for enhanced CTL attraction
Source: J Immunother Cancer. 2015 Mar 24;3:6. doi: 10.1186/s40425-015-0050-8 (PMC4371844; doi:10.1186/s40425-015-0050-8)
Supplement: Additional file 1: Figure S1. — Intratumoral CXCL8 expression shows strong correlation with the neutrophil marker, NCF2. Spearman (rho) analysis of the correlation between intra-tumoral mRNA expression of CXCL8 (IL-8) with Intra-tumoral expression of NCF2 (Neutrophil marker). Figure S2. BCG dose titration analysis and its impact on chemokine secretion by macrophages. Macrophages, generated by culturing monocytes in presence of GMCSF for 6 days, were exposed to increasing doses of BCG (0.4, 2, 10 × 106 CFU) for 24 hrs. Secretion of CXCL8 and CXCL10 was measured by specific ELISAs. Figure S3. BCG combination with IFNα + poly-I:C reduces CXCL10 levels in co-cultures of bladder cancer cells, macrophages and fibroblasts. TS4(Bladder cell line) was co cultured with macrophages and Fibroblasts in ratio 1:1:1 and treated with different permutations of celecoxib, IFNα, poly-I:C, and BCG. CXCL10 secretion was measured in 24 hour supernatants by ELISA. CCL22 and CXCL8 were below detection levels. [file 40425_2015_50_MOESM1_ESM.pdf]

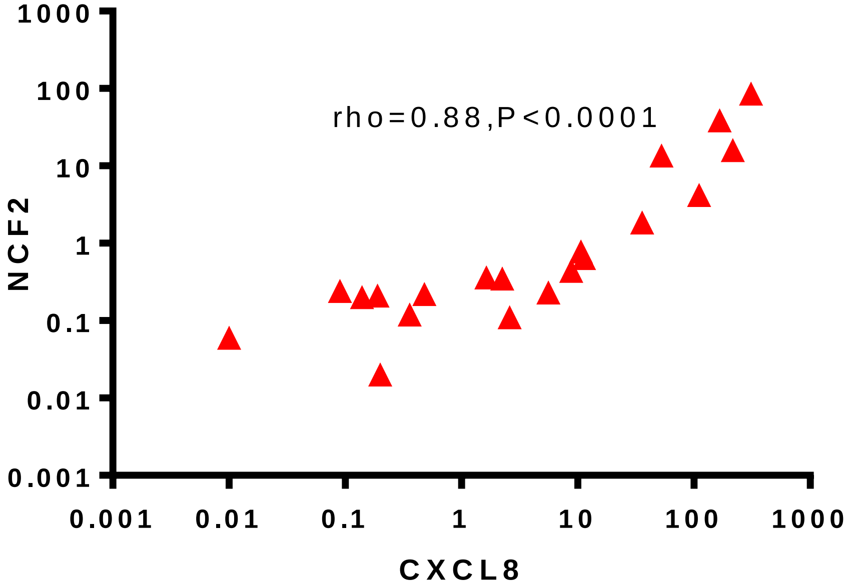

Supplementary Figure-1. Intratumoral CXCL8 expression shows strong correlation with the neutrophil marker, NCF2. Spearman (rho) analysis of the correlation between intra-tumoral mRNA expression of CXCL8 (IL-8) with Intra-tumoral expression of NCF2 (Neutrophil marker)

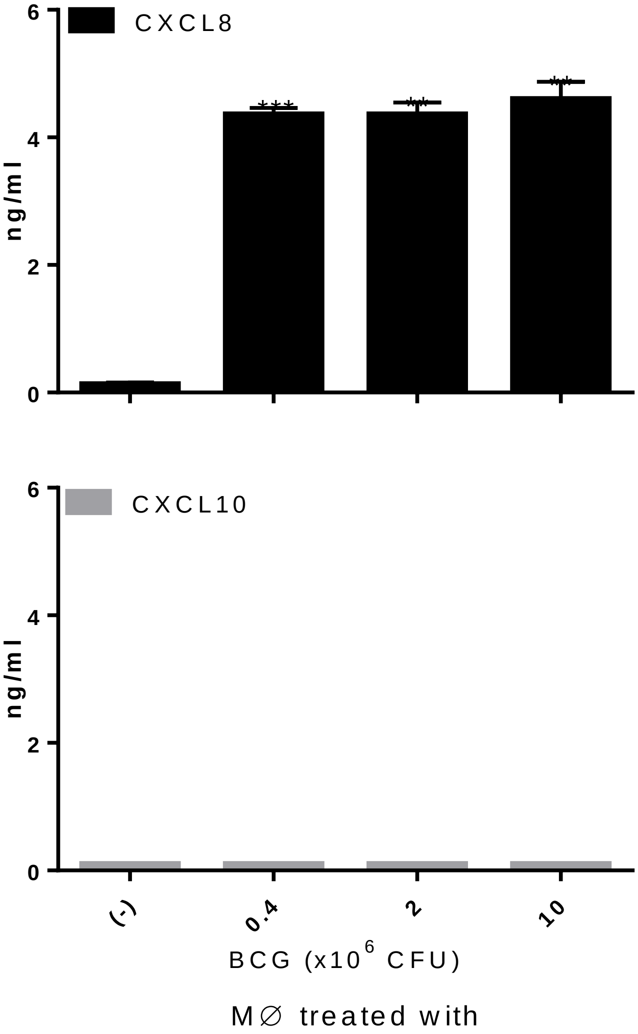

Supplementary Figure-2. BCG dose titration analysis and its impact on chemokine secretion by macrophages. Macrophages, generated by culturing monocytes in presence of GM-CSF for 6 days, were exposed to increasing doses of BCG (0.4, 2 , 10 x10^6 CFU) for 24hrs. Secretion of CXCL8 and CXCL10 was measured by specific ELISAs.

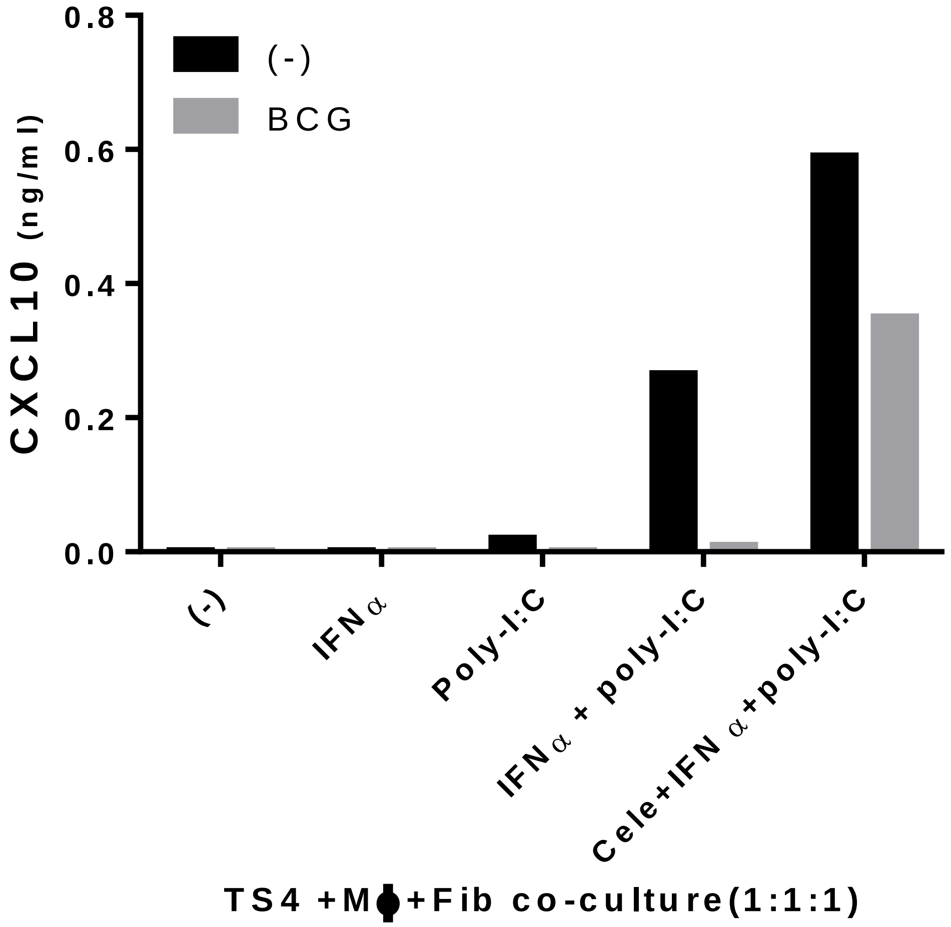

Supplementary Figure-3. BCG combination with IFNα + poly-I:C reduces CXCL10 levels in co-cultures of bladder cancer cells, macrophages and fibroblasts. TS4(Bladder cell line) was co cultured with macrophages and Fibroblasts in ratio 1:1:1 and treated with different permutations of celecoxib, IFNα, poly-I:C, and BCG. CXCL10 secretion was measured in 24 hour supernatants by ELISA. CCL22 and CXCL8 were below detection levels.
